# Lack of concern about body image and health during pregnancy linked to excessive gestational weight gain and small-for-gestational-age deliveries: the Japan Environment and Childrens Study

**DOI:** 10.1186/s12884-021-03827-0

**Published:** 2021-05-21

**Authors:** Naw Awn J-P, Marina Minami, Masamitsu Eitoku, Nagamasa Maeda, Mikiya Fujieda, Narufumi Suganuma, the Japan Environment and Childrens Study (JECS) Group, Michihiro Kamijima, Shin Yamazaki, Yukihiro Ohya, Reiko Kishi, Nobuo Yaegashi, Koichi Hashimoto, Chisato Mori, Shuichi Ito, Zentaro Yamagata, Hidekuni Inadera, Takeo Nakayama, Hiroyasu Iso, Masayuki Shima, Youichi Kurozawa, Narufumi Suganuma, Koichi Kusuhara, Takahiko Katoh

**Affiliations:** 1 Department of Environmental Medicine, Kochi Medical School, Kochi University, Nankoku, Kochi 783-8505 Japan; 2 Department of Obstetrics and Gynecology, Kochi Medical School, Kochi University, Nankoku, Kochi Japan; 3 Department of Pediatrics, Kochi Medical School, Kochi University, Nankoku, Kochi Japan

**Keywords:** Attitudes on gestational weight gain, Dieting, Overweight, Pregnancy, Small for gestational age, Underweight

## Abstract

**Background:**

Pregnant women in Japan express various reasons for limiting gestational weight gain (GWG). We aimed to identify and characterise groups where the women share common reasons to limit GWG and to examine how these groups are associated with inappropriate GWG and abnormal foetal size.

**Methods:**

We prospectively studied information from the Japan Environment and Childrens Study (JECS) on 92,539 women who gave birth to live singletons from 2011 through 2014. Pregnant women were recruited during early pregnancy. Their reasons for limiting GWG and other information were collected through self-reported questionnaires and medical records. We applied latent class analysis to group the women based on their reported reasons. We used multinomial logistic regression to compare the risks of inappropriate (inadequate and excessive) GWG and abnormal foetal size (determined by new-born weight for gestational age) between the identified groups.

**Results:**

We identified three groups: Group 1 (76.7%), concerned about delivery and new-born health (health-conscious women); Group 2 (14.5%), concerned about body shape, delivery, and new-born health (body-shape- and health-conscious women); and Group 3 (8.8%), women without strong reasons to limit GWG (women lacking body-shape and health consciousness). Compared with Group 1 members, Group 2 members tended to be younger, have lower pre-pregnancy weight, be unmarried, be nulliparous, have practiced weight loss before pregnancy, and not have chronic medical conditions. Group 3 members tended to be less educated, unmarried, multiparous, smokers, and have a higher prevalence of pre-pregnancy underweight and previous caesarean delivery. Relative to Group 1, Group 2 had a lower unadjusted risk for inadequate GWG (relative risk ratio [RRR]=0.86, 95% CI: 0.810.90) and large-for-gestational-age birth (RRR=0.91, 95% CI 0.860.97), whereas Group 3 had a higher unadjusted risk for excessive GWG (RRR=1.36, 95% CI: 1.291.43) and small-for-gestational-age (SGA) births (RRR=1.15, 95% CI: 1.051.25).

**Conclusions:**

In this Japanese nationwide birth cohort study, pregnant women who were less conscious about body shape and health had complex risks for excessive GWG and SGA birth. Health care providers should consider a womans perception of GWG when addressing factors affecting GWG and foetal growth.

**Supplementary Information:**

The online version contains supplementary material available at 10.1186/s12884-021-03827-0.

## Background

Women with a normal body weight when they conceive and who gain the recommended gestational weight have fewer adverse pregnancy and birth outcomes than those who do not [1,3]. Unlike European countries and the United States, where maternal obesity is a health concern [4], the proportion of underweight women of childbearing age (i.e.*,* body mass index [BMI] lower than 18.5kg/m^2^) is increasing in Japan [5]. Moreover, pregnant women in Japan believe that it is important to limit weight gain during pregnancy [6, 7]. Between 1980 and 2010, the prevalence of underweight women in Japan increased from 13.1 to 29.0% among 2029year olds and from 7.9 to 14.4% among 3039year olds [8]. A large-scale study examined 97,157 pregnant women who were registered in the Japan Society of Obstetrics and Gynecology registry system in 2013, and found that underweight women accounted for 18.2% of the total, whereas overweight (BMI=2529.9kg/m^2^) and obese (BMI 30kg/m^2^) women constituted 7.7 and 2.9%, respectively [2]. One recent Japanese study asked 1691 normal or underweight pregnant women about their perceived ideal gestational weight gain (GWG), and found that over 50% of the women thought that it should be kept below 12kg [6]. According to the Japanese recommendation [9], weight gain approximating 12kg is the upper limit for underweight and normal-weight pregnant women; however, the United States Institute of Medicine guideline [10] has this as the lower limit. The authors of the above study [6] also noted that pregnant women who reported a lower perceived ideal GWG gained less absolute weight during pregnancy and delivered smaller new-borns.

Studies conducted in Japan reported the presence of excessive concerns about body image and the practice of unnecessary weight control among young women [11, 12] and pregnant women [13] alike. These women might view weight gain during pregnancy as unpleasant and might worry about post-delivery body shape. However, a womans worries about her post-delivery body shape may not be the only issue surrounding her concern about GWG. In the recent survey of 1691 normal-weight or underweight pregnant women [6], the most common aspects mentioned were the ease of delivery and their own health and that of their new-born.

A better understanding of womens reasons for limiting GWG, including associated characteristics, and of any connection with inappropriate GWG and abnormal foetal size could help identify women at risk and enable health care providers to offer appropriate health guidance. We hypothesized the existence of latent groups formed by pregnant women who share common reasons to limit GWG, and that the womens underlying characteristics determined these reasons. The aims of our study were (1) to identify and characterise the latent groups where women share common reasons to limit GWG and (2) to examine the association of the identified groups with inappropriate GWG and abnormal foetal size among mothers of live-born singletons in the Japan Environment and Childrens Study (JECS).

## Methods

### Study design and setting

In this study, we used a dataset (jecs-an-20180131) from a nationwide prospective birth cohort study, the JECS. The detailed protocol and baseline information of participants have been reported previously [14, 15]. Briefly, around 100,000 pregnant women who live in 15 designated Study Areas were recruited during early pregnancy at obstetric facilities or at local government offices between January 2011 and March 2014. Participants will be followed until the participating children reach 13years of age. Eligibility was considered if a pregnant woman was (1) residing in a Study Area at the time of recruitment and expected to reside continually in Japan; (2) expected to give birth after August 1, 2011; and (3) capable of understanding the Japanese language and completing the self-administered questionnaires. The JECS collected demographic data and clinical and obstetric information through self-administered questionnaires or medical records transcripts. The questionnaires were distributed during the first trimester (first wave) and during the second/third trimester (second wave). The JECS protocol was approved by the Institutional Review Board on Epidemiological Studies of the Ministry of the Environment and by the Ethics Committees of all participating institutions. Written informed consent was obtained from all participants. If a woman was unmarried and younger than 20years, the consent was obtained from their parent or guardian.

### Study population

The data set comprised a total of 104,065 foetal records. For our analyses, we only considered participants who reported reasons for their perceived GWG. Next, we excluded participants based on predetermined exclusion criteria related to our outcomes of interest: gestational age<22weeks or>42weeks, or missing data; abortion, stillbirth, or missing birth status; multiple gestations or missing gestation status; missing birth weight; undetermined or missing sex of new-born; missing parity status; implausible maternal weight measured before delivery; and new-born with any chromosomal or major structural abnormality.

### Pregnant womens reasons to limit gestational weight gain

In the second wave of questionnaires, participants were asked to report their perceived ideal GWG as a range or a limit in kilograms. The questionnaires also asked how seriously they considered limiting weight gain during pregnancy so as not to exceed their perceived ideal GWG, and the reasons behind this perception. Response items for the reasons to limit GWG were (1) to deliver a healthy baby, (2) to have a smooth delivery, (3) to quickly restore pre-pregnancy body shape, (4) to avoid pregnancy stretch marks, (5) to avoid lifestyle diseases later in life, (6) to follow advice from health care providers, (7) to follow advice from family and friends, (8) no particular reason, and (9) other reasons. Responses to more than one item were possible. The original questionnaire (English language version) can be found in the Supplementary Figure (AdditionalFile1). We omitted two response items from the analyses: no particular reason (due to low response rate, 0.7%) and other reasons (because the number of similar responses was insufficient to generate an additional specific item).

### Outcome measurements

Our study had two main outcomes: inappropriate GWG and abnormal foetal size. We calculated GWG by subtracting self-reported pre-pregnancy weight from the last measured weight closest to delivery and categorized it as inadequate, appropriate, or excessive GWG, within each pre-pregnancy BMI stratum. Because the JECS recruited the women in early pregnancy, their pre-pregnancy weight measurements were not available. Therefore, we collected information regarding pre-pregnancy weight from questionnaires, interviews, and medical records and used the best available data. When stratifying GWG, we used the recommendations issued by the Ministry of Health, Labour and Welfare on appropriate GWG. The recommended GWG range for underweight pregnant women was 9 to 12kg; for normal-weight pregnant women was 7 to 12kg [9]; and for overweight pregnant women, 5 to 7kg. (The last was adapted from the Japanese Society for the Study of Obesity guidelines for GWG of 7kg in overweight and5kg in obese pregnant women). To determine foetal size, a new-borns birth weight (in grams) and gestational age at delivery (in days) were retrieved from medical records. Gestational age was determined by the ultrasound examinations performed during the first trimester or by the last menstrual period. We calculated new-born birth weight by gestational age, accounting for foetal sex and maternal parity [16]. We categorized foetal size as small for gestational age (SGA), appropriate for gestational age (AGA), or large for gestational age (LGA) as new-born birth weight below the 10th percentile, 10th to 90th percentile, or above the 90th percentile, respectively.

### Other variables

Maternal characteristics such as marital status, smoking habits and alcohol consumption, daily physical activity, and past medical history were obtained through the first wave of questionnaires. Education, prior weight loss practices, energy intake during pregnancy, and nausea and vomiting during pregnancy were obtained through the second wave of questionnaires. Information on maternal age, height and weight, parity, previous caesarean delivery, and receipt of health guidance was retrieved from medical records.

Daily physical activity during pregnancy was obtained by using the Japanese short version of the International Physical Activity Questionnaires, which considers all types of activities, including work-related, household chores, and leisure-time activities [17]. We calculated metabolic equivalent (MET) minutes per day (MET-min/d) and categorized it into three physical activity levels (tertiles). Participants were asked about weight loss practices before pregnancy; possible responses were reducing the size of their meals to two-thirds or less than normal, reducing snacks or midnight snacks, dieting, using a medication, purging after meals, smoking to lose weight, or exercising to lose weight. Multiple responses were possible and we categorized them by collapsing the responses: none (no weight loss practices), healthy methods (which included eating less or reducing snacks, dieting, and exercising to lose weight), and unhealthy methods (which included using a medication, purging after meals, and smoking to lose weight). Daily energy intake during pregnancy (kcal/d) was calculated based on the information collected through self-reported food frequency questionnaires [18] and used to form three groups (tertiles) with approximately equal number of participants. To account for potential physiological and psychosocial variations due to age, maternal age was divided into three groups: younger than 20years, 2034years, and 35years and older. Pre-pregnancy BMI was calculated as self-reported pre-pregnancy weight in kilograms divided by height in meters squared and stratified into underweight (<18.5kg/m^2^), normal weight (18.524.9kg/m^2^), and overweight (25kg/m^2^) in accordance with the Guidelines for Obstetrical Practice in Japan [19]. Smoking habit was categorized into three groups: never smoked, quit (before or after getting pregnant), or currently smoke. Other factors considered to influence the womens reasons to limit GWG were marital status (married or single [never married, divorced, or widowed]), educational level (high school or less, vocational school/college, university or higher), parity (0, 1, 2 or more), alcohol consumption (never, quit, currently drink), history of medical conditions (anaemia, chronic hypertension, diabetes mellitus), previous caesarean delivery, and receipt of pregnancy health guidance.

### Statistical analysis

We used latent class analysis, which provides a data-driven approach to identify latent groups (in our study, groups of women where members share common reasons to limit GWG) and the corresponding probability of each observation (pregnant woman) falling into each group. Models were repeatedly tested using different indicator variables (reasons to limit GWG) and specifying varying numbers of groups. Because we have only seven indicator variables, we tested models specifying two to four groups. The best model was determined based on Akaikes information criterion (AIC), Bayesian information criterion (BIC), and entropy [20, 21]. Smaller AIC and BIC indicate a better model, whereas entropy values close to 1.0 indicate a clear delineation of the groups.

**Fig. 1:**
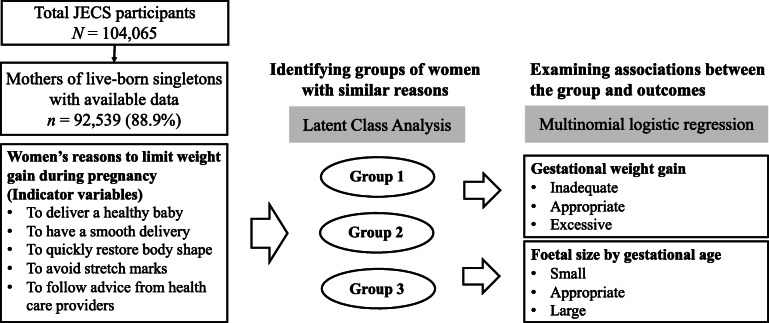
Schematic illustration of the study. The study question was whether women with different reasons to limit weight gain during pregnancy differ in their patterns of weight gain and fetal size. JECS=the Japan Environment and Childrens Study

Because our interest was to characterise the womens groups and to examine how these groups were associated with inappropriate GWG and abnormal foetal size, we conducted the following analyses. The association of maternal characteristics with the probability of being in each group of women was determined by using multinomial logistic regression. Group differences concerning maternal characteristics were examined by one-way ANOVA (followed by the Bonferroni correction for multiple comparisons) for continuous variables and the chi-squared test for categorical variables. We then constructed crude and adjusted multinomial logistic regression models to assess the associations between groups and inappropriate GWG (appropriate GWG as the reference group) or abnormal foetal size (AGA as the reference group). In the adjusted models, we included the following maternal characteristics considered to be the determinants of group membership and the outcomes: maternal age, pre-pregnancy BMI, marital status, parity, educational level, weight loss methods, total energy intake, physical activity, smoking and alcohol consumption habits, past medical history, previous caesarean delivery, pregnancy-related nausea and vomiting, and receipt of health guidance. Considering the well-known association between GWG and foetal size, we continued the analysis by adding GWG to the adjusted model that examined the association between the group and foetal size. Results were reported as crude and adjusted relative risk ratio (RRR) with 95% confidence intervals (CI). We also calculated the adjusted risk differences with 95% CI. A two-tailed *p*-value of <0.05 was considered statistically significant. All analyses were performed using Stata/MP 15.1 software (StataCorp., College Station, TX, USA). Figure1 illustrates the design of our study.

## Results

**Fig. 2:**
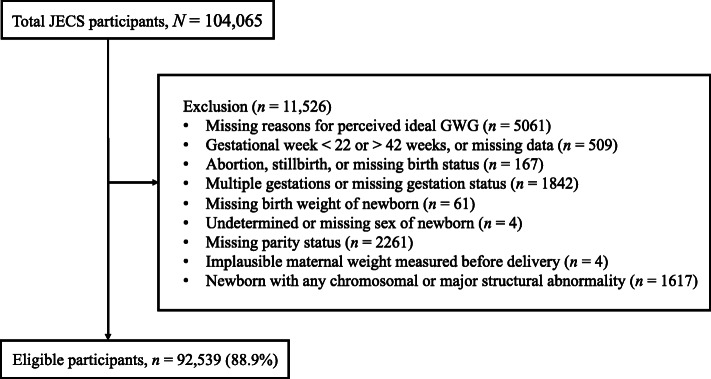
Schematic illustration of subject selection. JECS=Japan Environment and Childrens Study; GWG=gestational weight gain

Of the 104,065 foetal records that we reviewed, 5061 were excluded because no reasons for their perceived ideal GWG were given. An additional 6465 participants were excluded because they met the predetermined exclusion criteria related to our outcomes of interest (Fig.2). Thus, a total of 92,539 motherinfant pairs of live-born singletons (88.9% of the total) were included in our study.

### Latent class analysis

In our study, the most commonly reported reasons to limit GWG were to have a smooth delivery (72.7%), to deliver a healthy baby (68.9%), and to quickly restore pre-pregnancy body shape (46.7%) (Table1). Latent class models that included all the indicator variables (reasons to limit GWG) and were tested for four latent groups failed to stratify distinct groups. Model fitness measures are presented in Supplemental Table 1 (AdditionalFile1). The main model identified three groups of women and were named according to the groups common reasons (Table2): Group 1 (health conscious, 76.7%), smooth delivery and bearing a healthy child were common issues; Group 2 (body-shape and health conscious, 14.5%), concerns about ones own body shape, delivery, and health of the child were shared reasons; and Group 3 (neither body-shape nor health conscious, 8.8%), members were less likely to possess any strong reason. Table3 presents maternal characteristics associated with members of Groups 2 and 3, relative to women in Group 1. A pregnant woman who was younger, had lower BMI, and was single (including those who were divorced or widowed) was more likely to be a member of Group 2 or 3. However, women in Group 2 tended to be nulliparous, practice weight loss before becoming pregnant, drink alcohol, and not have chronic medical conditions, whereas women in Group 3 were more likely to be parous, not engage in weight loss practices, be less educated, smoke during pregnancy, and have had a previous caesarean delivery.

**Table 1:** Proportion of women reporting each reason (*N*=92,539)

Reasons to limit gestational weight gain	*n* (%)
To have a smooth delivery	67,303 (72.7)
To deliver a healthy baby	63,798 (68.9)
To quickly restore body shape	43,191 (46.7)
To avoid stretch marks	14,255 (15.4)
To avoid lifestyle diseases later in life	27,081 (29.3)
To follow advice from health care providers	26,451 (28.6)
To follow advice from family and friends	7819 (8.5)
No particular reason	619 (0.7)
Other reasons	4643 (5.0)

No particular reason and Other reasons were omitted from the analyses because of the low response (0.7%) and inability to generate a meaningful item, respectively

**Table 2:** Probability of giving each reason by group (*N*=92,539)

	Group 1	Group 2	Group 3
	71,002 (76.7%)	13,406 (14.5%)	8131 (8.8%)
Reasons to limit gestational weight gain	Probability (95% CI)		
To deliver a healthy baby	0.72 (0.710.72)	0.80 (0.790.81)	0.27 (0.240.30)
To have a smooth delivery	0.79 (0.780.79)	0.88 (0.870.89)	
To quickly restore body shape	0.42 (0.410.44)	0.90 (0.880.93)	0.08 (0.060.10)
To avoid stretch marks	0.03 (0.020.04)	0.83 (0.690.91)	
To follow advice from health care providers	0.29 (0.280.29)	0.38 (0.370.39)	0.13 (0.110.14)

Groups were defined on the basis of the distribution of reasons to limit gestational weight gain: Group 1=more likely to cite smooth delivery and child health (health conscious); Group 2=more likely to cite body shape, smooth delivery, and child health (body-shape and health conscious); Group 3=less likely to cite any strong reason (not body-shape or health conscious)

Women in Group 3 had a very low probability of citing to have a smooth delivery and to avoid stretch marks; therefore, we constrained those two items in the model for Group 3

**Table 3:** Maternal characteristics determining membership in a particular group, relative to Group 1

	Group 2				Group 3			
Maternal factors	RRR	95% CI	aRRR	95% CI	RRR	95% CI	aRRR	95% CI
Maternal age, y								
19 (teenage mother)	1.47	1.261.73	1.06	0.891.27	1.90	1.582.28	1.51	1.221.86
2034	Ref.		Ref.		Ref.		Ref.	
35	0.59	0.560.62	0.69	0.650.72	0.87	0.820.92	0.87	0.820.92
BMI categories, kg/m^2^								
<18.5 (underweight)	1.17	1.121.23	1.23	1.171.30	1.33	1.261.42	1.22	1.141.30
18.524.9 (normal weight)	Ref.		Ref.		Ref.		Ref.	
25 (overweight)	0.41	0.380.44	0.41	0.370.44	1.06	0.991.14	0.95	0.881.03
Marital status								
Married	Ref.		Ref.		Ref.		Ref.	
Single mother	1.48	1.361.60	1.10	1.011.21	1.58	1.431.75	1.30	1.151.45
Educational level								
High school or less	Ref.		Ref.		Ref.		Ref.	
Vocational school/College	1.01	0.971.05	1.04	0.991.09	0.64	0.610.68	0.75	0.700.79
University or higher	0.88	0.840.93	0.91	0.860.96	0.57	0.540.61	0.73	0.680.78
Weight loss methods^a^								
None	Ref.		Ref.		Ref.		Ref.	
Healthy method	1.36	1.311.41	1.38	1.331.45	0.67	0.640.70	0.71	0.670.75
Unhealthy method	2.19	2.052.33	2.09	1.942.25	1.05	0.961.15	0.83	0.750.91
Total energy intake, kcal/d								
1st (lowest tertile)	1.04	1.001.09	0.98	0.941.03	1.18	1.121.25	1.07	1.011.14
2nd	Ref.		Ref.		Ref.		Ref.	
3rd	1.02	0.981.07	1.03	0.991.08	1.17	1.111.24	1.15	1.081.22
Physical activity, MET-min/d								
1st (lowest tertile)	0.97	0.921.01	0.98	0.931.03	1.07	1.011.13	1.01	0.951.08
2nd	Ref.		Ref.		Ref.		Ref.	
3rd	1.14	1.091.20	1.06	1.011.12	1.18	1.121.26	1.10	1.041.17
Smoking								
Never smoke	Ref.		Ref.		Ref.		Ref.	
Quit	1.12	1.081.17	1.03	0.991.08	1.50	1.421.57	1.38	1.311.46
Currently smoke	1.22	1.111.33	1.10	0.991.22	2.77	2.543.02	2.15	1.952.38
Alcohol								
Never drink	Ref.		Ref.		Ref.		Ref.	
Quit	1.20	1.151.25	1.12	1.081.17	1.04	0.991.10	0.99	0.941.05
Currently drink	1.06	0.991.13	1.12	1.041.20	0.97	0.891.06	0.97	0.881.06
Parity								
0	Ref.		Ref.		Ref.		Ref.	
1	0.68	0.660.71	0.76	0.730.80	1.20	1.141.27	1.16	1.091.23
2 or more	0.53	0.500.56	0.60	0.570.64	1.34	1.261.42	1.21	1.131.30
Past medical history								
Anaemia	1.04	0.991.09			1.03	0.971.09		
Hypertension	0.46	0.390.54	0.67	0.560.79	1.23	1.081.40	1.13	0.981.30
Diabetes	0.57	0.450.73	0.87	0.681.11	1.11	0.891.39	1.04	0.821.33
Previous caesarean delivery	0.70	0.650.76	0.97	0.901.06	1.51	1.411.63	1.44	1.321.56
Nausea and vomiting	0.99	0.941.04	1.05	1.001.11	0.89	0.840.95	0.93	0.870.99
Health guidance	0.89	0.830.94	0.92	0.860.98	1.01	0.941.08	0.99	0.911.07

*BMI* body mass index, *CI* confidence interval, *RRR* crude relative risk ratio, *aRRR* adjusted relative risk ratio, *MET* metabolic equivalent

Group 1=health conscious; Group 2=body-shape and health conscious; Group 3=not body-shape or health conscious

^a^Healthy method=eating less or reducing snacks, dieting, or exercising to lose weight; Unhealthy method=using medication, purging after meals, or smoking to lose weight

Adjusted model included age, pre-pregnancy body mass index, marital status, parity, educational level, weight loss methods, total energy intake, physical activity, smoking and alcohol consumption habit, past medical history (hypertension, diabetes), previous caesarean delivery, pregnancy-related nausea and vomiting, and receipt of health guidance

### Characteristics and study outcomes of the womens groups

Group 1 (health conscious) generally reflected the overall populations characteristics, except they tended to be older, more educated, and non-smoking (Table4). Group 2 (body-shape and health conscious) were younger, had a lower average pre-pregnancy weight, tended to practice weight loss before pregnancy, were more likely to be unmarried and nulliparous, and less likely to have past medical conditions. Group 3 (not body-shape or health conscious) had relatively higher proportions of teenagers and pre-pregnancy underweight and women who were less educated, smokers, parous, had a history of hypertension, or had undergone previous caesarean delivery, but fewer women who engaged in weight loss practices. Of the 92,539 pregnant women, 17.7% were classified as having inadequate GWG and 33.9% as having excessive GWG; 7.5% had an SGA birth and 10.1% an LGA birth. Group 2 comprised the population with the lowest proportion of women with inadequate GWG (15.9%) and LGA births (9.3%), and Group 3 comprised the population with the highest proportion of women with excessive GWG (39.8%) and SGA birth (8.4%).

**Table 4:** Maternal characteristics and study outcomes, overall and by group

	All	Group 1	Group 2	Group 3	
	*n* (%)				*P*^*a*^
	92,539	71,002 (76.7)	13,406 (14.5)	8131 (8.8)	
Age, y, mean (SD)	30.7 (5.0)	31.0 (5.0)	29.4 (5.0)***	30.2 (5.3)***	<0.001
Age categories, y					<0.001
19 (teenage mother)	998 (1.1)	651 (0.9)	202 (1.5)	145 (1.8)	
2034	67,786 (73.2)	51,051 (71.9)	10,744 (80.1)	5991 (73.7)	
35	21,943 (23.7)	17,889 (25.2)	2231 (16.6)	1823 (22.4)	
Missing	1812 (2.0)				
BMI, kg/m^2^, mean (SD)	21.2 (3.3)	21.4 (3.4)	20.5 (2.5)***	21.2 (3.5)***	<0.001
BMI categories, kg/m^2^					<0.001
<18.5 (underweight)	14,867 (16.1)	10,805 (15.2)	2502 (18.7)	1560 (19.2)	
18.524.9 (normal weight)	67,712 (73.2)	51,871 (73.1)	10,229 (76.3)	5612 (69.0)	
25 (overweight)	9914 (10.7)	8290 (11.7)	671 (5.0)	953 (11.7)	
Missing	46 (0.05)				
Educational level					<0.001
High school or less	33,571 (36.3)	25,019 (35.2)	4835 (36.1)	3717 (45.7)	
Vocational school/College	38,695 (41.8)	29,989 (42.2)	5838 (43.5)	2868 (35.3)	
University or higher	19,735 (21.3)	15,709 (22.1)	2686 (20.0)	1340 (16.5)	
Missing	538 (0.6)				
Weight loss methods^b^					<0.001
None	39,072 (42.2)	30,360 (42.8)	4550 (33.9)	4162 (51.2)	
Healthy method	46,612 (50.4)	35,985 (50.7)	7330 (54.7)	3297 (40.5)	
Unhealthy method	6855 (7.4)	4657 (6.6)	1526 (11.4)	672 (8.3)	
Total energy intake, kcal/d					<0.001
1st (lowest tertile)	30,925 (33.4)	23,538 (33.1)	4536 (33.8)	2851 (35.1)	
2nd	30,807 (33.3)	23,929 (33.7)	4425 (33.0)	2453 (30.2)	
3rd	30,794 (33.3)	23,523 (33.1)	4444 (33.1)	2827 (34.8)	
Missing	13 (0.01)				
Physical activity, MET-min/d					<0.001
1st (lowest tertile)	31,986 (34.6)	24,853 (35.0)	4390 (32.7)	2743 (33.7)	
2nd	28,475 (30.8)	22,149 (31.2)	4040 (30.1)	2286 (28.1)	
3rd	29,901 (32.3)	22,463 (31.6)	4692 (35.0)	2746 (33.8)	
Missing	2177 (2.3)				
Smoking					<0.001
Never smoke	53,212 (57.5)	41,910 (59.0)	7503 (56.0)	3799 (46.7)	
Quit	33,834 (36.6)	25,315 (35.6)	5087 (37.9)	3432 (42.2)	
Currently smoke	4392 (4.7)	2990 (4.2)	651 (4.9)	751 (9.2)	
Missing	1101 (1.2)				
Alcohol					<0.001
Never drink	31,764 (34.3)	24,794 (34.9)	4198 (31.3)	2772 (34.1)	
Quit	50,790 (54.9)	38,495 (54.2)	7808 (58.2)	4487 (55.2)	
Currently drink	9151 (9.9)	7104 (10.0)	1275 (9.5)	772 (9.5)	
Missing	834 (0.9)				
Single mother	3887 (4.2)	2682 (3.8)	732 (5.5)	473 (5.8)	<0.001
Parity					<0.001
0	37,260 (40.3)	27,743 (39.1)	6760 (50.4)	2757 (33.9)	
1	36,078 (39.0)	28,047 (39.5)	4680 (34.9)	3351 (41.2)	
2 or more	19,201 (20.7)	15,212 (21.4)	1966 (14.7)	2023 (24.9)	
Past medical history					
Anaemia (yes)	17,184 (18.6)	13,101 (18.4)	2551 (19.0)	1532 (18.8)	0.135
Hypertension (yes)	2301 (2.5)	1873 (2.6)	166 (1.2)	262 (3.2)	<0.001
Diabetes (yes)	875 (0.9)	708 (1.0)	77 (0.6)	90 (1.1)	<0.001
Previous caesarean delivery (yes)	7622 (8.2)	5851 (8.2)	798 (5.9)	973 (12.0)	<0.001
Nausea and vomiting (yes)	76,326 (82.5)	58,780 (82.8)	11,080 (82.6)	6466 (79.5)	<0.001
Health guidance (yes)	9877 (10.7)	7689 (10.8)	1300 (9.7)	888 (10.9)	0.001
GWG, kg, mean (SD)	10.3 (4.0)	10.2 (4.0)	10.7 (3.8) ***	10.8 (4.5) ***	<0.001
GWG categories					<0.001
Inadequate	16,405 (17.7)	12,872 (18.1)	2126 (15.9)	1407 (17.3)	
Appropriate	42,976 (46.4)	33,210 (46.8)	6410 (47.8)	3356 (41.3)	
Excessive	31,362 (33.9)	23,550 (33.2)	4574 (34.1)	3238 (39.8)	
Missing	1796 (1.9)				
Birth weight, g, mean (SD)	3030.8 (408.2)	3035.0 (406.9)	3018.5 (399.0)***	3013.8 (433.0)***	<0.001
Foetal size					0.001
SGA	6973 (7.5)	5250 (7.4)	1043 (7.8)	680 (8.4)	
AGA	76,261 (82.4)	58,533 (82.4)	11,110 (82.9)	6618 (81.4)	
LGA	9305 (10.1)	7219 (10.2)	1253 (9.3)	833 (10.2)	

*AGA* appropriate for gestational age, *BMI* body mass index, *GWG* gestational weight gain, *LGA* large for gestational age, *SGA* small for gestational age

Group 1=health conscious; Group 2=body-shape and health conscious; Group 3=not body-shape or health conscious

^a^ *p*-values from chi-squared test or one-way ANOVA

^b^ Healthy method=eating less or reducing snacks, dieting, or exercising to lose weight; Unhealthy method=using medication, purging after meals, or smoking to lose weight

Bonferroni multiple comparisons (compared to Group 1): *<0.05, **<0.01, ***<0.001

Average GWG and new-born birth weight according to maternal characteristics are presented in Table5.

**Table 5:** Average gestational weight gain (GWG) and new-born birth weight by maternal characteristics

	GWG, kg		Birth weight, g	
	Mean (SD)	*P*^a^	Mean (SD)	*P*^a^
Overall participants	10.3 (4.0)		3030.8 (408.2)	
Maternal age, y		< 0.001		< 0.001
19 (teenage mother)	11.9 (4.5)***		2994.3 (377.7)**	
2034 (Reference)	10.5 (4.0)		3034.1 (401.0)	
35	9.6 (3.9)***		3021.8 (431.6)***	
BMI categories, kg/m^2^		< 0.001		< 0.001
<18.5	10.9 (3.5)***		2930.7 (386.3)***	
18.524.9 (Reference)	10.6 (3.7)		3038.9 (400.2)	
25	7.7 (5.3)***		3125.2 (461.7)***	
GWG categories				< 0.001
Inadequate	4.8 (2.9)		2858.1 (445.2)***	
Appropriate (Reference)	9.6 (1.5)		3009.4 (375.6)	
Excessive	14.1 (2.9)		3154.7 (387.1)***	
Marital status		< 0.001		< 0.001
Married	10.2 (4.0)		3032.7 (408.1)	
Single mother	11.6 (4.5)		2997.0 (410.9)	
Parity		< 0.001		< 0.001
0 (Reference)	10.7 (4.1)		2999.0 (409.2)	
1	10.0 (3.9)***		3044.4 (397.6)***	
2 or more	10.0 (4.0)***		3066.9 (421.4)***	
Educational level		< 0.001		0.158
High school or less (Reference)	10.7 (4.4)		3030.0 (413.5)	
Vocational school/College	10.2 (3.8)***		3029.3 (408.5)	
University or higher	9.8 (3.5)***		3035.8 (397.3)	
Weight loss methods^b^		< 0.001		< 0.001
None (Reference)	10.2 (3.7)		3018.8 (405.9)	
Healthy method	10.2 (4.1)**		3041.5 (409.1)***	
Unhealthy method	11.5 (4.8)***		3026.1 (413.5)	
Total energy intake, kcal/d		< 0.001		< 0.001
1st (lowest tertile)	10.0 (4.1)***		3015.4 (408.7)***	
2nd (Reference)	10.3 (3.9)		3032.2 (407.5)	
3rd	10.6 (4.0)***		3044.8 (408.0)***	
Physical activity, MET-min/d		< 0.001		0.004
1st (lowest tertile)	10.2 (4.0)***		3026.1 (407.9)**	
2nd (Reference)	10.1 (3.9)		3037.2 (408.2)	
3rd	10.6 (4.1)***		3031.3 (407.6)	
Smoking		< 0.001		< 0.001
Never smoke (Reference)	9.8 (3.7)		3030.3 (404.0)	
Quit	10.9 (4.3)***		3046.6 (411.3)***	
Currently smoke	11.1 (4.7)***		2922.0 (420.3)***	
Alcohol		< 0.001		< 0.001
Never drink (Reference)	10.1 (4.0)		3024.0 (408.7)	
Quit	10.5 (4.0)***		3034.4 (407.6)**	
Currently drink	9.9 (3.9)***		3037.0 (410.7)*	
Anaemia		0.015		< 0.001
No	10.3 (4.0)		3028.2 (408.4)	
Yes	10.4 (3.9)		3042.4 (408.0)	
Hypertension		< 0.001		< 0.001
No	10.3 (4.0)		3034.0 (404.9)	
Yes	9.2 (4.7)		2909.3 (511.6)	
Diabetes		< 0.001		0.089
No	10.3 (4.0)		3030.7 (407.5)	
Yes	7.4 (5.5)		3054.3 (488.8)	
Previous caesarean delivery		< 0.001		< 0.001
No	10.4 (4.0)		3043.1 (409.0)	
Yes	9.6 (4.1)		2893.5 (372.9)	
Nausea and vomiting		< 0.001		< 0.001
No	10.8 (3.9)		3007.0 (426.0)	
Yes	10.2 (4.0)		3035.7 (404.0)	
Health guidance		0.613		< 0.001
No	10.3 (3.9)		3027.6 (404.4)	
Yes	10.3 (4.8)		3067.5 (425.3)	

*BMI* body mass index, *GWG* gestational weight gain

^a^ *p*-values are from one-way ANOVA or Students t-tests

^b^ Healthy method=eating less or reducing snacks, dieting, or exercising to lose weight; Unhealthy method=using medication, purging after meals, or smoking to lose weight

Bonferroni correction for multiple comparisons (compared to reference category): *<0.05, **<0.01, ***<0.001

### Association of groups with inappropriate GWG and abnormal foetal size

The average GWGs were higher for Groups 2 and 3 than for Group 1 (10.24.0kg in Group 1 vs. 10.73.8kg and 10.84.5kg, respectively, for Groups 2 and 3; all *p*<0.001), but the average birth weights were lower in Groups 2 and 3 (3035.0406.9g in Group 1 vs. 3018.5399.0g and 3013.8433.0g, respectively, in Groups 2 and 3; all *p*<0.001) (Table4). In the multinomial logistic regression analyses, Group 2 showed a reduced risk for inadequate GWG (RRR=0.86, [95% CI: 0.810.90]) relative to Group 1, whereas Group 3 showed a higher risk for excess GWG (RRR=1.36, 95% CI: 1.291.43) when appropriate GWG was used as the reference category. Adjusting for the maternal characteristics determining group membership lessened the associations (Table6). Relative to Group 1, Group 2 showed a reduced risk for LGA births (RRR=0.91; 95% CI: 0.860.97), whereas Group 3 showed an increased risk for SGA birth (RRR=1.15, 95% CI: 1.051.25), when specifying AGA as the reference category. Adjusting for the maternal characteristics determining group membership erased the associations. However, adjusting for GWG, in addition to the other maternal characteristics, elevated the risk of SGA birth in Group 3 (Table7). The adjusted risk differences are provided in Supplemental Table2 (AdditionalFile1). Compared with Group 1, Group 2 had 9 fewer women per 1000 women with inadequate GWG, whereas Group 3 had 48 more women per 1000 women with excessive GWG. Compared with Group 1, Group 3 had 7 more women per 1000 women with an SGA birth. Crude and adjusted relative risk ratios for inappropriate GWG and abnormal foetal size according to maternal characteristics can be found in Supplemental Table3 (AdditionalFile1) and Supplemental Table4 (AdditionalFile1), respectively.

**Table 6:** Relative risk ratio for inappropriate GWG, relative to Group 1

	Group 1 (*n*=71,002)	Group 2 (*n*=13,406)					Group 3 (*n*=8131)				
	Cases, *n* (%)	Cases, *n* (%)	RRR	95% CI	aRRR	95% CI	Cases, *n* (%)	RRR	95% CI	aRRR	95% CI
**GWG**											
Inadequate	12,872 (18.1)	2126 (15.9)	0.86	0.810.90	0.93	0.880.98	1407 (17.3)	1.08	1.011.15	1.07	0.991.15
Appropriate	33,210 (46.8)	6410 (47.8)	Ref.		Ref.		3356 (41.3)	Ref.		Ref.	
Excessive	23,550 (33.2)	4574 (34.1)	1.01	0.961.05	0.98	0.941.03	3238 (39.8)	1.36	1.291.43	1.28	1.211.35

*CI* confidence interval, *RRR* crude relative risk ratio, *aRRR* adjusted relative risk ratio, *GWG* gestational weight gain

Group 1=health conscious; Group 2=body-shape and health conscious; Group 3=not body-shape or health conscious

Adjusted maternal characteristics include age, pre-pregnancy body mass index, marital status, parity, educational level, weight loss methods, total energy intake, physical activity, smoking and alcohol consumption habit, past medical history (anaemia, hypertension, diabetes), previous caesarean delivery, pregnancy-related nausea and vomiting, and receipt of health guidance

**Table 7:** Relative risk ratio for adverse foetal size, relative to Group 1

	Group 1 (*n*=71,002)	Group 2 (*n*=13,406)							Group 3 (*n*=8131)						
	Cases, *n* (%)	Cases, *n* (%)	RRR	95% CI	aRRR^1^	95% CI	aRRR^2^	95% CI	Cases, *n* (%)	RRR	95% CI	aRRR^1^	95% CI	aRRR^2^	95% CI
**Foetal size**															
SGA	5250 (7.4)	1043 (7.8)	1.05	0.981.12	1.03	0.961.11	1.04	0.971.12	680 (8.4)	1.15	1.051.25	1.08	0.991.19	1.11	1.011.22
AGA	58,533 (82.4)	11,110 (82.9)	Ref.		Ref.		Ref.		6618 (81.4)	Ref.		Ref.		Ref.	
LGA	7219 (10.2)	1253 (9.3)	0.91	0.860.97	0.97	0.911.04	0.96	0.901.03	833 (10.2)	1.02	0.951.10	1.07	0.981.16	1.02	0.941.11

*CI* confidence interval, *RRR* crude relative risk ratio, *aRRR* adjusted relative risk ratio, *AGA* appropriate for gestational age, *LGA* large for gestational age, *SGA* small for gestational age

Group 1=health conscious; Group 2=body-shape and health conscious; Group 3=not body-shape or health conscious

aRRR^1^: Adjusted maternal characteristics included age, pre-pregnancy body mass index, marital status, parity, educational level, weight loss methods, total energy intake, physical activity, smoking and alcohol consumption habit, past medical history (anaemia, hypertension, diabetes), previous caesarean delivery, pregnancy-related nausea and vomiting, and receipt of health guidance

aRRR^2^: Adjusted for gestational weight gain in addition to maternal characteristics adjusted in aRRR^1^

## Discussion

In this Japanese cohort of pregnant women who gave birth to live singletons between 22 and 42weeks of gestation, three groups were identified based on shared reasons to limit GWG. We investigated the association between group classification and inappropriate GWG or abnormal foetal size. The traditional approach is to link individual risk factors with these outcomes, making our approach unique. In our study, women who were body-shape and health conscious appeared to gain more weight during pregnancy; however, the higher weight gain put them at no elevated risk for inappropriate GWG or abnormal foetal size. We also found that women lacking body-shape and health consciousness gained more weight and were at a higher risk for excessive GWG; surprisingly, they were also at a higher risk for SGA birth.

Women in Group 1, who wanted to have a smooth delivery and to bear a healthy child, were likely to be more health conscious than those in the other groups. This group made up three-fourths of the women, and generally reflected the characteristics of the women overall. The groups prevalence of inappropriate GWG and of abnormal foetal size were also comparable to those of the overall study population. Group 1 also served as a comparison to highlight the distinct characteristics that put women at a higher risk for inappropriate GWG and abnormal foetal size.

We found that lower pre-pregnancy body weight represented no additional risk for abnormal foetal size in healthy young Japanese pregnant women. Compared with the health-conscious women, women who were both body-shape and health conscious gained more weight, and their risk for inadequate GWG was lower. Their underlying characteristics, such as younger age and previous weight loss practices, might have contributed to a higher weight gain, which is supported by past reports [2, 3, 22]. It is possible that women who practiced weight loss before pregnancy stopped this practice, overate during pregnancy, or both, and therefore gained more weight; however, the risk for abnormal foetal size did not differ from that of health-conscious women (Group 1).

Women who showed a lack of concern about body shape and health during pregnancy (Group 3) were at a higher risk for excessive GWG, and their foetuses were at risk of SGA. On average, they gained more absolute weight during pregnancy but delivered lighter babies than health-conscious women. The underlying characteristics of these women included being teenagers, having less education, being unmarried, being parous, having a pre-pregnancy weight at the high and low extremes, smoking, suffering from hypertension, and having undergone previous caesarean delivery. High pre-pregnancy BMI and stopping smoking have previously been associated with excessive GWG [23]. Parity was inversely associated with GWG in our cohort; however, a recent meta-analysis reported mixed findings across studies [24]. Increased risk for excessive GWG seen in teenage mothers and pregnant single mothers could be due to poor eating habits and lower dietary quality, such as eating mostly processed foods or an energy-dense diet, particularly with added sugar and fat and less protein [25]. After adjusting for these factors, the risk of excessive GWG remained higher in the women lacking concern about body shape and health during pregnancy than in the health-conscious group. We suggest that psychosocial factors, not investigated in our study, could have played an important role in GWG, although the association between psychosocial factors and GWG remains unclear. One recent systematic review reported a direct association between excessive GWG and body image dissatisfaction, depression, and a lower level of social support [26]. However, another similar review stated that depression was not related to excessive GWG; instead, the authors reported a negative weight gain attitude and less knowledge about GWG were risk factors for excessive GWG [27]. There is also potential for variation in the GWG between rural and urban dwellers [28], or across geographic regions [29], factors that we did not examine in our study. Maternal factors such as pre-pregnancy underweight, smoking, and hypertension have been reported to increase the risk of SGA [2, 30,32]. Lower maternal education is associated with delayed foetal growth, leading to lower foetal weight and SGA foetuses [33, 34]. Adjusting for these maternal factors erased the between-group differences in SGA risk, indicating that modification of maternal factors reduces SGA birth. However, controlling for GWG, in addition to the above maternal factors, put these women back to an elevated risk for SGA birth, signifying the need to tailor GWG individually based on the mothers characteristics.

Less than 11% of the women in the study reported that they received pregnancy health guidance. This information deserves special attention from the system that delivers health care to mothers and children. In Japan, the delivery of pregnancy health guidance mainly focuses on limiting weight gain in overweight or obese women to avoid risks during pregnancy and delivery [7]. Because both biological factors (e.g., preterm and SGA births) and social factors (e.g., teenage and single mothers) are associated with increased perinatal mortality [35, 36], it is essential to provide health guidance to all pregnant women, particularly those with the complex risk factors associated with Group 3 in this study. In this subgroup of women, we observed that, for every 1000 women, 48 more women had excessive GWG and 7 more women had an SGA birth when compared to the majority. In our Japanese cohort of pregnant women, about one-third had not exceeded high school education. Ones educational level determines ones general and health-related knowledge, literacy, occupation, and income [34]. Low maternal education was associated with a higher risk for preterm birth and impaired foetal growth [33, 34]. Multiple health risks such as smoking, alcohol abuse, and unhealthy diet are more prevalent among teenage mothers [37], the less educated [34], and low-income populations [38]. Being unmarried or having medical conditions might provoke psychological distress when added to the stress of being pregnant. Thus, it is possible that psychosocial conditions prevent healthy behaviours from occurring. Pregnant women see their health care providers as the most reliable source of information and are expecting guidance from their clinicians [39, 40]. Pregnancy causes many biological and physical changes in women, which may lead to varying attitudes toward weight gain. Some women in Japan see weight gain as a good sign that their baby is growing healthily; however, others may worry that their baby will become too large. Some women view physical changes in pregnancy as attractive, but others might view them as unpleasant [7, 13]. Women may not be aware of their appropriate weight gain range and the actual consequences of excessive or inadequate GWG. Sufficient antenatal education regarding the ill effects of excessive or inadequate GWG on the health of the mother and foetus could raise awareness of appropriate GWG, thus leading to better pregnancy and birth outcomes.

Our study is the first to uncover a group of women who were less conscious of body shape and health, their corresponding characteristics, and their associated risks for inappropriate GWG and abnormal foetal size in a large Japanese cohort. The availability of a wide range of information on these Japanese women as part of the JECS enabled us to identify important characteristics associated with excessive GWG and SGA birth. There are several potential limitations to our study. First, the questionnaire used to identify the womens reasons to limit GWG was not validated. However, each response item was constructed with a clear definition for each reason studied. We found that the distribution of the responses to the items in our study was comparable to that in a previous study that used a similar questionnaire [6], and the items were adequate for identifying distinct groups. Second, we restricted our study to those women who reported reasons to limit GWG; because the questionnaire was distributed after the first trimester, this may have excluded less healthy women and unfavourable pregnancies, and may have resulted in possible underestimation of the risks. Moreover, the weight loss practices and proportion of underweight women might be different in different populations. Third, some womens concerns about weight gain and body shape may have arisen from an awareness of increased food intake or weight gain after conception (during the first trimester) [22], which may affect their responses and hence the group members. However, we found that a womans underlying characteristics, such as age, parity, pre-pregnancy body weight, educational status, and previous weight loss practicefactors unaffected by conception or trimesterswere the main determinants of group membership and study outcomes. Fourth, self-reported pre-pregnancy weight was used to calculate GWG and pre-pregnancy BMI, which may have resulted in under- or overestimation. Although under- or over-reporting is possible, self-reported pre-pregnancy weight shows a strong correlation with measured weight, indicating that the ranking of individuals was well-preserved [41], and allowing for unbiased risk estimation for excessive or inadequate GWG [42]. Fifth, we defined GWG categories using appropriate GWG cut-offs recommended for Japanese pregnant women, which are lower than those of the Institute of Medicine guidelines [10]; this should be considered in generalizing the results to other populations. Finally, we did not have information on whether participants exerted any effort to control their weight gain during pregnancy, but we did adjust for daily energy intake and physical activity information in our analyses.

## Conclusion

In this Japanese cohort of pregnant women who gave birth to live singletons, three groups of women were identified who shared common reasons to limit GWG. These groups were identified to test potential factors explaining inappropriate GWG and abnormal foetal size risks. Compared with the majority, healthy young women who practiced weight loss before pregnancy showed concern about body shape and health during pregnancy; they gained more weight and were at a lower risk for inadequate GWG, but no elevated risk for LGA birth. In contrast, women lacking consciousness about body shape and health were at a higher risk for excessive GWG, whereas their foetuses were at risk of SGA. These findings highlight the importance of considering a pregnant womans view of GWG for identifying underlying factors that put her at a higher risk for inappropriate GWG and the delivery of a foetus of abnormal weight.

## Supplementary Information

**Additional file 1: Supplemental Figure.** Original English language version of the questionnaire on the respondents reasons to limit gestational weight gain. **Supplemental Table1.** Goodness-of-fit measures for different models of latent class analyses, *N*=92,539. **Supplemental Table2.** Adjusted risk difference for the outcomes GWG and foetal size, compared to Group 1. **Supplemental Table3.** Association between maternal characteristics and inappropriate GWG, relative to appropriate-for-gestation weight gain. **Supplemental Table4.** Association between maternal characteristics and abnormal foetal size, relative to appropriate-for-gestational-age group.

## Data Availability

Data are unsuitable for public deposition because of ethical considerations and restrictions as per legal framework of Japan. It is prohibited by the Act on the Protection of Personal Information (Act No. 57 of 30 May 2003, amended on 9 September 2015) to publicly deposit data containing personal information. Ethical Guidelines for Medical and Health Research Involving Human Subjects, enforced by the Japan Ministry of Education, Culture, Sports, Science and Technology and the Ministry of Health, Labour and Welfare, also restricts the open sharing of epidemiologic data. All inquiries about access to data should be addressed Dr. Shoji F. Nakayama, JECS Programme Office, National Institute for Environmental Studies, at jecs-en@nies.go.jp.

## Abbreviations

AGA: Appropriate for gestational age
AIC: Akaikes information criterion
BIC: Bayesian information criterion
BMI: Body mass index
GWG: Gestational weight gain
JECS: Japan Environment and Childrens Study
LGA: Large for gestational age
MET-min/d: Metabolic equivalent minutes per day
RRR: Relative risk ratios
SGA: Small for gestational age
