# Supplementary material for: Lack of concern about body image and health during pregnancy linked to excessive gestational weight gain and small-for-gestational-age deliveries: the Japan Environment and Childrens Study
Source: BMC Pregnancy Childbirth. 2021 May 21;21:396. doi: 10.1186/s12884-021-03827-0 (PMC8139142; doi:10.1186/s12884-021-03827-0)
Supplement: Supplementary file 1 — Additional file 1: Supplemental Figure. Original English language version of the questionnaire on the respondents reasons to limit gestational weight gain. Supplemental Table1. Goodness-of-fit measures for different models of latent class analyses, N=92,539. Supplemental Table2. Adjusted risk difference for the outcomes GWG and foetal size, compared to Group 1. Supplemental Table3. Association between maternal characteristics and inappropriate GWG, relative to appropriate-for-gestation weight gain. Supplemental Table4. Association between maternal characteristics and abnormal foetal size, relative to appropriate-for-gestational-age group. [file 12884_2021_3827_MOESM1_ESM.docx]

**Title:** Lack of concern about body image and health during pregnancy linked to excessive gestational weight gain and small-for-gestational-age deliveries: the Japan Environment and Children’s Study

**Author names:** Naw Awn J-P^1^, Marina Minami^1^, Masamitsu Eitoku^1^, Nagamasa Maeda^2^, Mikiya Fujieda^3^, Narufumi Suganuma^1^, and the Japan Environment and Children’s Study (JECS) Group

^1^Department of Environmental Medicine, Kochi Medical School, Kochi University, Nankoku, Kochi, Japan

^2^Department of Obstetrics and Gynecology, Kochi Medical School, Kochi University, Nankoku, Kochi, Japan

^3^Department of Pediatrics, Kochi Medical School, Kochi University, Nankoku, Kochi, Japan

**Additional file 1**

**Supplemental Figure** Original English language version of the questionnaire on the respondent’s reasons to limit gestational weight gain

**
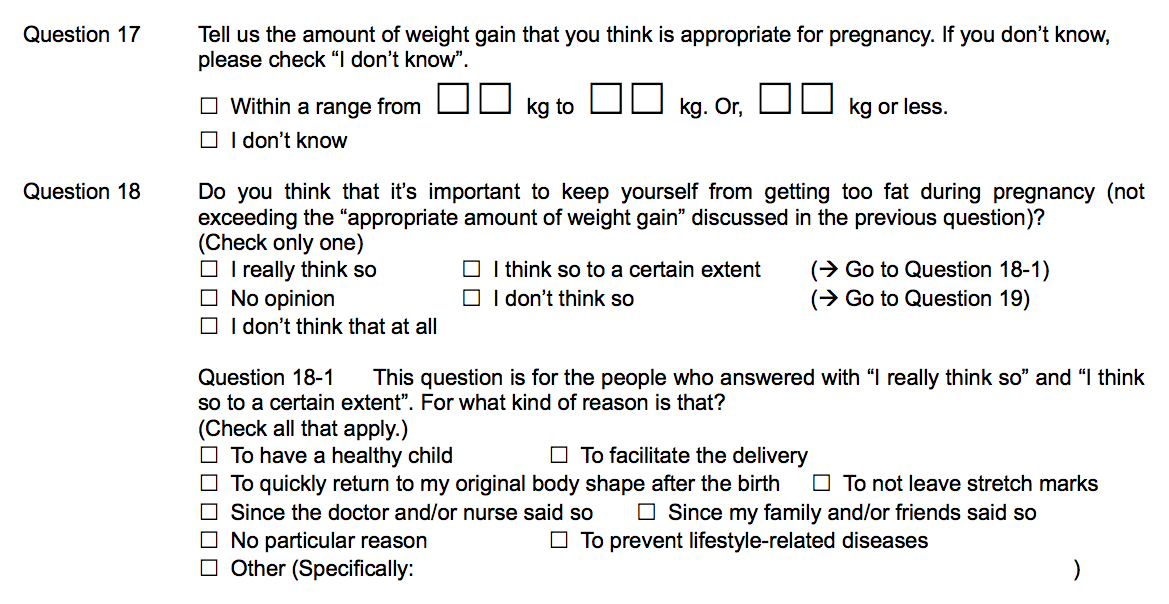
**

**Supplemental Table 1** Goodness-of-fit measures for different models of latent class analyses, *N* = 92,539

| Model | BIC | AIC | Entropy |
| --- | --- | --- | --- |
| 2-class model^a^ | 526245.5 | 526141.7 | 0.49 |
| 2-class model^b^ | 636076.1 | 635953.5 | 0.50 |
| 3-class model^a^ | 523826.1 | 523684.6 | 0.61 |
| 3-class model^b^ | 632495.2 | 632334.8 | 0.53 |

AIC = Akaike’s information criterion; BIC = Bayesian information criterion

^a^ models used the following indicator variables: to deliver a healthy baby, to have a smooth delivery, to quickly restore body shape, to avoid pregnancy stretch marks, and to follow advice from health care providers

^b^ model used “to avoid lifestyle diseases later in life” in addition to the indicator variables used in the ^a^ model

2-class = model specifying 2 latent groups; 3-class = model specifying 3 latent groups

Smaller AIC and BIC indicate a better model, whereas entropy values closer to 1.0 indicate clearer delineation of the groups

The 3-class model^a^ was chosen as the main model

**Supplemental Table 2** Adjusted risk difference for the outcomes GWG and foetal size, with Group 1 as the referent

|  | Group 1 | Group 2 |  |  | Group 3 |  |  |
| --- | --- | --- | --- | --- | --- | --- | --- |
|  | Predicted risk | Predicted risk | aRD^a^ | 95% CI | Predicted risk | aRD^a^ | 95% CI |
| **GWG** |  |  |  |  |  |  |  |
| Inadequate | 18.26 | 17.36 | –0.009 | –0.016 to –0.002 | 17.68 | –0.006 | –0.015 to 0.003 |
| Appropriate | 47.84 | 48.71 | 0.009 | –0.001 to 0.018 | 43.58 | –0.043 | –0.054 to –0.031 |
| Excessive | 33.9 | 33.92 | 0.0003 | –0.009 to 0.009 | 38.73 | 0.048 | 0.037 to 0.06 |
| **Foetal size** |  |  |  |  |  |  |  |
| SGA | 7.34 | 7.63 | 0.003 | –0.002 to 0.008 | 8.05 | 0.007 | 0.001 to 0.014 |
| AGA | 82.6 | 82.64 | 0.001 | –0.007 to 0.008 | 81.74 | –0.009 | –0.018 to 0.001 |
| LGA | 10.06 | 9.72 | –0.003 | –0.009 to 0.002 | 10.21 | 0.002 | –0.006 to 0.009 |

CI = confidence interval; aRD = adjusted risk difference; GWG = gestational weight gain; AGA = appropriate for gestational age; LGA = large for gestational age; SGA = small for gestational age

Group 1 = health conscious; Group 2 = body-shape and health conscious; Group 3 = not body-shape or health conscious

^a^Adjusted maternal characteristics include age, pre-pregnancy body mass index, marital status, parity, educational level, weight loss methods, total energy intake, physical activity, smoking and alcohol consumption habit, past medical history (anaemia, hypertension, diabetes), previous caesarean delivery, pregnancy-related nausea and vomiting, and receipt of health guidance. Gestational weight gain was additionally adjusted in the models for the foetal size outcome.

**Supplemental Table 3** Association between maternal characteristics and inappropriate GWG, relative to appropriate-for-gestation weight gain

|  | Inadequate GWG | |  |  | Excessive GWG | |  |  |
| --- | --- | --- | --- | --- | --- | --- | --- | --- |
|  | RRR | 95% CI | aRRR | 95% CI | RRR | 95% CI | aRRR | 95% CI |
| Maternal age, y |  |  |  |  |  |  |  |  |
| ≤19 (teenage mother) | 0.97 | 0.79–1.18 | 1.00 | 0.80–1.26 | 1.73 | 1.51–1.99 | 1.15 | 0.98–1.34 |
| 20–34 | Ref. |  | Ref. |  | Ref. |  | Ref. |  |
| ≥35 | 1.20 | 1.15–1.25 | 1.21 | 1.15–1.26 | 0.74 | 0.71–0.77 | 0.79 | 0.76–0.82 |
| BMI categories, kg/m^2^ |  |  |  |  |  |  |  |  |
| <18.5 | 2.75 | 2.62–2.88 | 2.86 | 2.72–3.00 | 1.53 | 1.47–1.60 | 1.53 | 1.46–1.60 |
| 18.5–24.9 | Ref. |  | Ref. |  | Ref. |  | Ref. |  |
| ≥25 | 6.08 | 5.70–6.49 | 5.63 | 5.25–6.03 | 5.60 | 5.28–5.93 | 5.29 | 4.97–5.62 |
| Single mother (married as reference) | 0.89 | 0.81–0.99 | 0.89 | 0.79–0.99 | 1.76 | 1.64–1.89 | 1.26 | 1.16–1.36 |
| Parity |  |  |  |  |  |  |  |  |
| 0 | Ref. |  | Ref. |  | Ref. |  | Ref. |  |
| 1 | 1.06 | 1.02–1.11 | 1.01 | 0.97–1.06 | 0.78 | 0.76–0.81 | 0.80 | 0.77–0.83 |
| 2 or more | 1.08 | 1.02–1.13 | 0.99 | 0.95–1.06 | 0.84 | 0.81–0.87 | 0.80 | 0.77–0.84 |
| Educational level |  |  |  |  |  |  |  |  |
| High school or less | Ref. |  | Ref. |  | Ref. |  | Ref. |  |
| Vocational school/College | 0.95 | 0.91–0.99 | 0.98 | 0.94–1.03 | 0.68 | 0.66–0.70 | 0.80 | 0.77–0.83 |
| University or higher | 0.96 | 0.92–1.01 | 1.02 | 0.97–1.08 | 0.50 | 0.48–0.53 | 0.68 | 0.65–0.71 |
| Weight loss methods^a^ |  |  |  |  |  |  |  |  |
| None | Ref. |  | Ref. |  | Ref. |  | Ref. |  |
| Healthy method | 0.95 | 0.91–0.98 | 1.02 | 0.98–1.07 | 1.22 | 1.18–1.26 | 1.14 | 1.10–1.18 |
| Unhealthy method | 1.00 | 0.92–1.08 | 1.12 | 1.02–1.22 | 2.22 | 2.10–2.36 | 1.46 | 1.37–1.56 |
| Total energy intake, kcal/d |  |  |  |  |  |  |  |  |
| 1st (lowest tertile) | 1.14 | 1.09–1.19 | 1.11 | 1.06–1.16 | 0.99 | 0.96–1.03 | 0.88 | 0.85–0.91 |
| 2nd | Ref. |  | Ref. |  | Ref. |  | Ref. |  |
| 3rd | 0.91 | 0.87–0.96 | 0.90 | 0.85–0.94 | 1.15 | 1.11–1.19 | 1.13 | 1.09–1.17 |
| Physical activity, MET-min/d |  |  |  |  |  |  |  |  |
| 1st (lowest tertile) | 0.99 | 0.95–1.03 | 0.97 | 0.92–1.01 | 1.08 | 1.04–1.12 | 1.05 | 1.01–1.09 |
| 2nd | Ref. |  | Ref. |  | Ref. |  | Ref. |  |
| 3rd | 0.94 | 0.90–0.98 | 0.94 | 0.90–0.99 | 1.29 | 1.24–1.34 | 1.13 | 1.09–1.18 |
| Smoking habit |  |  |  |  |  |  |  |  |
| Never smoker | Ref. |  | Ref. |  | Ref. |  | Ref. |  |
| Quit | 0.88 | 0.84–0.91 | 0.86 | 0.83–0.90 | 1.71 | 1.66–1.76 | 1.52 | 1.47–1.57 |
| Current smoker | 1.12 | 1.02–1.22 | 1.01 | 0.92–1.13 | 2.24 | 2.09–2.39 | 1.68 | 1.55–1.81 |
| Alcohol drinking |  |  |  |  |  |  |  |  |
| Never | Ref. |  | Ref. |  | Ref. |  | Ref. |  |
| Quit | 0.88 | 0.85–0.92 | 0.93 | 0.89–0.97 | 1.08 | 1.05–1.12 | 0.97 | 0.93–1.00 |
| Current drinking | 0.92 | 0.87–0.98 | 0.95 | 0.89–1.02 | 0.85 | 0.81–0.90 | 0.85 | 0.80–0.90 |
| Past medical history |  |  |  |  |  |  |  |  |
| Anemia | 0.97 | 0.93–1.02 | 1.00 | 0.95–1.05 | 0.94 | 0.91–0.98 | 1.02 | 0.98–1.06 |
| Hypertension | 1.57 | 1.41–1.75 | 1.22 | 1.09–1.38 | 1.16 | 1.05–1.28 | 0.99 | 0.89–1.11 |
| Diabetes | 3.11 | 2.65–3.64 | 2.18 | 1.82–2.60 | 1.12 | 0.94–1.33 | 0.75 | 0.62–0.91 |
| Previous caesarean delivery | 1.17 | 1.10–1.24 | 1.04 | 0.97–1.12 | 0.87 | 0.82–0.92 | 0.86 | 0.81–0.92 |
| Nausea and vomiting (No as reference) | 1.24 | 1.18–1.31 | 1.25 | 1.18–1.32 | 0.88 | 0.85–0.91 | 0.90 | 0.86–0.94 |
| Health guidance | 1.33 | 1.26–1.41 | 1.20 | 1.13–1.27 | 1.41 | 1.34–1.47 | 1.26 | 1.20–1.33 |
| Child’s sex (male as reference) | 1.02 | 0.99–1.06 | – |  | 0.95 | 0.92–0.97 | – |  |
| Gestational week | 0.83 | 0.82–0.84 | – |  | 1.17 | 1.16–1.18 | – |  |
| Birth weight, g | 0.99 | 0.99–0.99 | – |  | 1.00 | 1.00–1.00 | – |  |

BMI = body mass index; CI = confidence interval; GWG = gestational weight gain; RRR = crude relative risk ratio; aRRR = adjusted relative risk ratio

^a^Healthy method = eating less or reducing snacks, dieting, or exercising to lose weight; Unhealthy method = using medication, purging after meals, or smoking to lose weight

Variables included in the multivariate model: age, marital status, parity, educational level, pre-pregnancy BMI, weight loss methods, total energy intake, physical activity, smoking and alcohol consumption habit, past medical history (anemia, hypertension, diabetes), previous caesarean delivery, pregnancy-related nausea and vomiting, and receipt of health guidance

**Supplemental Table 4** Association between maternal characteristics and abnormal foetal size, relative to appropriate-for-gestational-age group

|  | SGA |  |  |  | LGA |  |  |  |
| --- | --- | --- | --- | --- | --- | --- | --- | --- |
|  | RRR | 95% CI | aRRR | 95% CI | RRR | 95% CI | aRRR | 95% CI |
| Maternal age, y |  |  |  |  |  |  |  |  |
| ≤19 (teenage mother) | 1.15 | 0.92–1.44 | 0.97 | 0.75–1.27 | 0.99 | 0.80–1.22 | 0.87 | 0.69–1.11 |
| 20–34 | Ref. |  | Ref. |  | Ref. |  | Ref. |  |
| ≥35 | 1.05 | 0.99–1.11 | 1.05 | 0.98–1.11 | 1.14 | 1.08–1.19 | 1.18 | 1.12–1.25 |
| BMI categories, kg/m^2^ |  |  |  |  |  |  |  |  |
| <18.5 | 1.57 | 1.48–1.66 | 1.49 | 1.40–1.59 | 0.57 | 0.52–0.61 | 0.58 | 0.53–0.63 |
| 18.5–24.9 | Ref. |  | Ref. |  | Ref. |  | Ref. |  |
| ≥25 | 0.81 | 0.74–0.89 | 0.78 | 0.71–0.87 | 2.00 | 1.89–2.12 | 1.75 | 1.64–1.87 |
| GWG categories |  |  |  |  |  |  |  |  |
| Inadequate | 1.65 | 1.56–1.75 | 1.63 | 1.53–1.73 | 0.78 | 0.72–0.84 | 0.73 | 0.67–0.79 |
| Appropriate | Ref. |  | Ref. |  | Ref. |  | Ref. |  |
| Excessive | 0.62 | 0.59–0.67 | 0.61 | 0.57–0.65 | 2.11 | 2.01–2.21 | 1.97 | 1.87–2.08 |
| Single mother (married as reference) | 1.07 | 0.95–1.21 | 0.96 | 0.84–1.10 | 1.11 | 1.00–1.23 | 1.02 | 0.91–1.15 |
| Parity |  |  |  |  |  |  |  |  |
| 0 | Ref. |  | Ref. |  | Ref. |  | Ref. |  |
| 1 | 0.95 | 0.90–1.00 | 0.95 | 0.89–1.01 | 0.75 | 0.73–0.79 | 0.75 | 0.71–0.80 |
| 2 or more | 0.86 | 0.80–0.92 | 0.81 | 0.75–0.88 | 0.96 | 0.91–1.02 | 0.94 | 0.88–1.00 |
| Educational level |  |  |  |  |  |  |  |  |
| High school or less | Ref. |  | Ref. |  | Ref. |  | Ref. |  |
| Vocational school/College | 0.96 | 0.91–1.02 | 0.97 | 0.92–1.04 | 0.94 | 0.89–0.98 | 0.98 | 0.93–1.03 |
| University or higher | 0.90 | 0.84–0.96 | 0.87 | 0.81–0.94 | 0.91 | 0.86–0.97 | 1.03 | 0.96–1.10 |
| Weight loss methods^a^ |  |  |  |  |  |  |  |  |
| None | Ref. |  | Ref. |  | Ref. |  | Ref. |  |
| Healthy method | 0.93 | 0.88–0.97 | 1.02 | 0.96–1.08 | 1.20 | 1.15–1.26 | 1.03 | 0.98–1.08 |
| Unhealthy method | 0.99 | 0.91–1.10 | 1.05 | 0.95–1.17 | 1.28 | 1.18–1.39 | 1.04 | 0.95–1.14 |
| Total energy intake, kcal/d |  |  |  |  |  |  |  |  |
| 1st (lowest tertile) | 1.10 | 1.04–1.17 | 1.05 | 0.99–1.12 | 0.99 | 0.95–1.05 | 1.00 | 0.94–1.06 |
| 2nd | Ref. |  | Ref. |  | Ref. |  | Ref. |  |
| 3rd | 0.98 | 0.92–1.04 | 0.99 | 0.93–1.06 | 1.09 | 1.04–1.15 | 1.06 | 1.00–1.12 |
| Physical activity, MET–min/d |  |  |  |  |  |  |  |  |
| 1st (lowest tertile) | 0.96 | 0.90–1.02 | 0.95 | 0.89–1.01 | 0.93 | 0.88–0.98 | 0.93 | 0.88–0.99 |
| 2nd | Ref. |  | Ref. |  | Ref. |  | Ref. |  |
| 3rd | 0.99 | 0.93–1.06 | 0.99 | 0.93–1.06 | 0.99 | 0.94–1.04 | 0.95 | 0.90–1.00 |
| Smoking habit |  |  |  |  |  |  |  |  |
| Never smoker | Ref. |  | Ref. |  | Ref. |  | Ref. |  |
| Quit | 0.93 | 0.88–0.98 | 1.01 | 0.95–1.07 | 1.11 | 1.06–1.16 | 0.99 | 0.94–1.04 |
| Current smoker | 1.79 | 1.63–1.97 | 2.00 | 1.79–2.23 | 0.73 | 0.65–0.83 | 0.62 | 0.54–0.71 |
| Alcohol drinking |  |  |  |  |  |  |  |  |
| Never | Ref. |  | Ref. |  | Ref. |  |  |  |
| Quit | 0.97 | 0.92–1.03 | 0.97 | 0.92–1.03 | 1.04 | 0.99–1.09 | 1.02 | 0.97–1.07 |
| Current drinking | 0.99 | 0.91–1.08 | 1.00 | 0.91–1.10 | 1.02 | 0.95–1.11 | 1.03 | 0.95–1.12 |
| Past medical history |  |  |  |  |  |  |  |  |
| Anemia | 0.94 | 0.89–1.01 | 0.95 | 0.89–1.02 | 1.01 | 0.95–1.06 | 1.09 | 1.02–1.15 |
| Hypertension | 1.48 | 1.29–1.70 | 1.76 | 1.52–2.04 | 1.06 | 0.93–1.22 | 0.91 | 0.78–1.06 |
| Diabetes | 1.16 | 0.90–1.49 | 1.03 | 0.78–1.36 | 2.09 | 1.76–2.49 | 1.71 | 1.40–2.07 |
| Previous caesarean delivery | 0.74 | 0.67–0.82 | 0.73 | 0.65–0.81 | 0.98 | 0.91–1.06 | 1.02 | 0.93–1.11 |
| Nausea and vomiting (No as reference) | 0.82 | 0.77–0.87 | 0.83 | 0.77–0.88 | 1.04 | 0.98–1.11 | 1.10 | 1.03–1.17 |
| Health guidance | 0.90 | 0.83–0.98 | 0.90 | 0.82–0.98 | 1.45 | 1.36–1.54 | 1.28 | 1.20–1.37 |

BMI = body mass index; CI = confidence interval; GWG = gestational weight gain; LGA = large-for-gestational-age; RRR = crude relative risk ratio; aRRR = adjusted relative risk ratio; SGA = small-for-gestational-age

^a^Healthy method = eating less or reducing snacks, dieting, or exercising to lose weight; Unhealthy method = using medication, purging after meals, or smoking to lose weight

Variables included in the multivariate model: age, marital status, parity, educational level, pre-pregnancy BMI, gestational weight gain, weight loss methods, total energy intake, physical activity, smoking and alcohol consumption habit, past medical history (anemia, hypertension, diabetes), previous caesarean delivery, pregnancy-related nausea and vomiting, and receipt of health guidance
